# Three-Dimensional–Printed Vaginal Molds for Treating Postradiation Vaginal Stenosis

**DOI:** 10.1097/og9.0000000000000082

**Published:** 2025-05-29

**Authors:** B. Mitchell Bodily, Anton Edelmann, Kozdronkiewicz Michal, Allen Mehr

**Affiliations:** Obstetrics and Gynecology Residency Program, and the Department of Obstetrics and Gynecology, Tripler Army Medical Center, Honolulu, Hawaii.

## Abstract

**BACKGROUND:**

Pelvic radiation therapy for gynecologic cancers often results in vaginal stenosis, negatively affecting sexual function. Vaginal dilation therapy has low adherence rates. Three dimensional (3D)–printed vaginal molds offer an individualized, passive alternative to maintain vaginal length and prevent stenosis. These personalized molds can be worn comfortably for extended periods, require minimal effort, and can preserve vaginal caliber and length.

**CASE:**

A 38-year-old patient with stage IVB adenosquamous cervical carcinoma, treated with pelvic radiation, experienced complete vaginal stenosis. She underwent lysis of vaginal adhesions, with an individualized 3D-printed mold placed after surgery; she resumed normal sexual function 6 months posttreatment.

**CONCLUSION:**

A customized 3D-printed vaginal mold effectively maintained vaginal caliber and function after reconstructive surgery, increasing the patient’s quality of life and sexual function.

## Abstract 2

A three-dimensional–printed vaginal mold effectively maintained vaginal caliber after reconstructive surgery, increasing the patient's quality of life and sexual function.

**Teaching Points**

- Three-dimensional–printed molds offer a more comfortable, passive alternative to traditional vaginal dilation therapy, potentially leading to improved patient compliance and outcomes.
- Early intervention with customized molds may prevent the development of vaginal stenosis and improve long-term quality of life for patients undergoing pelvic radiation.
- Three-dimensional–printed molds allow for iterative design improvements based on patient feedback, which can enhance device function and improve patient comfort.

Vaginal stenosis, fibrosis, and loss of vaginal elasticity are common sequelae of pelvic radiation for treatment of gynecologic malignancy and may occur in more than 80% of patients.^1^ To avoid these negative outcomes, vaginal dilation therapy is recommended by experts and by the American Cancer Society, with use ranging from three 15-minute sessions two to three times weekly.^2,3^ Reports regarding adherence to vaginal dilation therapy show it to be as low as 25%, with barriers including pain, embarrassment, and a loss of modesty.^1,4^

## CASE

A 38-year-old patient was referred for treatment of vaginal stenosis secondary to radiation therapy. At age 29 years, she had experienced heavy vaginal bleeding while in labor, prompting a cesarean delivery. On further workup for persistent bleeding, she was found to have a 7-cm mass extending from the cervix to the left pelvic sidewall and upper vagina. Based on imaging and biopsy findings, she was diagnosed with stage IVB, poorly differentiated adenosquamous cervical carcinoma and started on radio-sensitizing cisplatin chemotherapy and external beam radiation therapy.

The patient received a total 5,840 centigray of external beam radiation therapy, 750 centigray of brachytherapy, and five cycles of cisplatin. She had a complete response to this therapy. After treatment, she developed severe vaginal stenosis, with shortening of the vaginal canal and dyspareunia. This complication prevented clinicians from being able to visualize the cervix on examination and interfered with the patient’s sexual function. Vaginal dilation was recommended. Unfortunately, the patient reported that the dilators were uncomfortable, belittling, and time-consuming. After several months of decreased use, she noted progressive difficulty with penetrative intercourse. This led her to use barriers between her and her partner, such as the “Ohnut Depth Limiting Ring,”^5^ to limit the depth of penetration during intercourse due to pain.

**Fig. 1.**
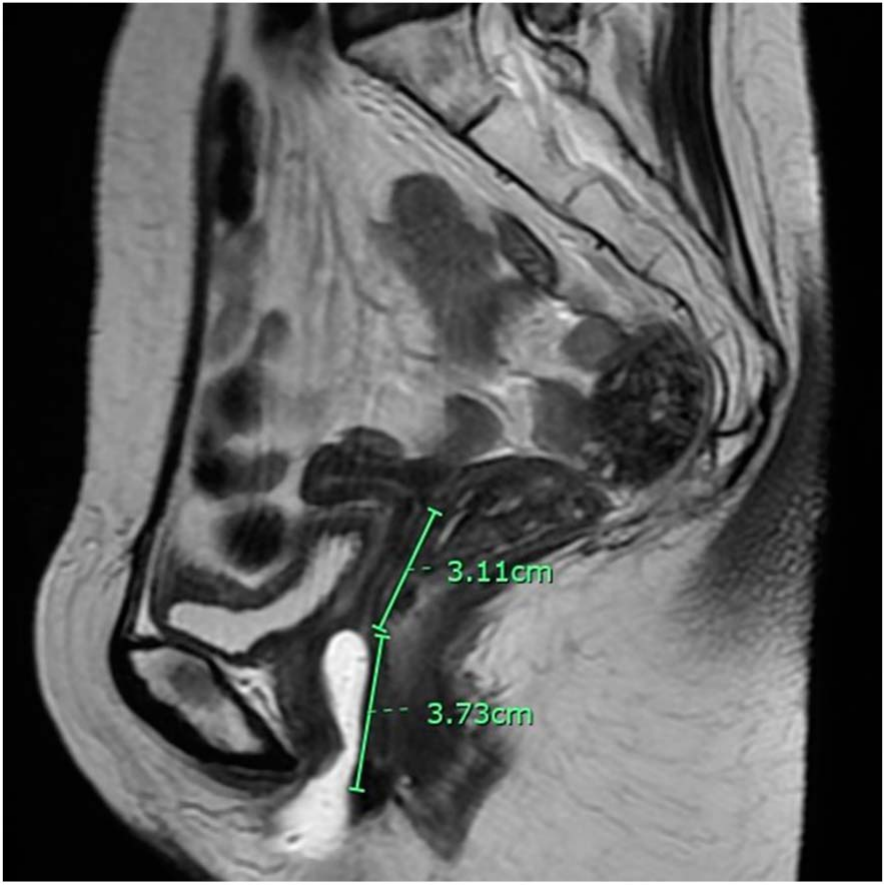
Sagittal T2-weighted magnetic resonance image showing 3.7-cm vagina length and 3.1-cm closed vaginal apex.

At initial presentation, the patient had a 3-cm vaginal length and diameter, with atrophic vaginal mucosa. The mid-portion of the vagina ended in a blind pouch with a small pinhead opening at the left side that was thought to be either the cervical remnant or a tract to the proximal vagina. Magnetic resonance imaging showed a 3.1-cm upper vaginal segment (Fig. 1).

*Bodily. 3D-Printed Vaginal Molds. O&G Open 2025*.

After careful counseling, the patient underwent reconstructive surgery. After lysis of adhesions and excision of a scarred transverse band, the vaginal dimensions were measured to be 7.5×3.5 cm. The upper vaginal epithelium was found to be well vascularized and did not require any grafts. A temporary mold was cut from foam used for patient positioning during laparoscopic surgeries; this technique has been described previously for McIndoe neovagina procedures using upholstery foam.^6^ The foam was cut to 1.5 times the measured vaginal dimensions and was placed inside a sterile ultrasound sheath and into the vagina to prevent vaginal adhesions until a three-dimensional (3D)–printed mold could be made.

The patient returned to the office 24 hours later for placement of the first mold. The initial mold was inspired by the Nichols-Counseller mold used after neovagina creation for patients with congenital anomalies such as Mayer-Rokitansky-Küster-Hauser syndrome.^7^ The patient was encouraged to wear the mold up to 18 hours per day while the vaginal mucosa was healing and to take it out as needed for a period of up to 6 hours for cleaning and comfort.

**Fig. 2.**
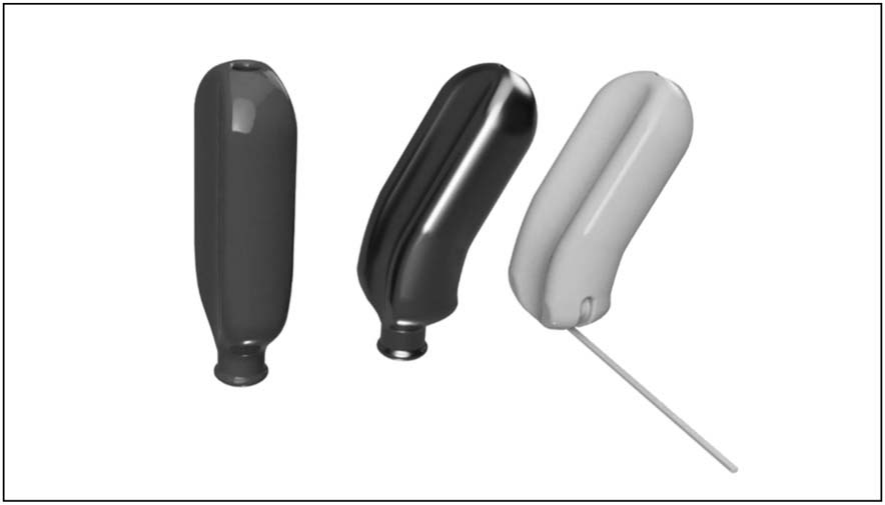
Progressive design modifications from left to right.

At her next follow-up appointment 72 hours later, the patient was having difficulty with emptying her bladder and a slow urinary stream, which required removal of the mold up to seven times per day to void. Given these symptoms, the mold was redesigned with an additional recess along the anterior portion to better conform to the H-shape of the vagina and to reduce pressure under the urethra and bladder neck to allow for voiding. After an additional week of wear, the patient provided feedback that the portion of the mold protruding past the hymen caused perineal irritation. With our final design, we removed the portion protruding past the hymen to improve comfort. However, given the need to be able to remove the mold to allow for cleaning, we added a 3D-printed string that could be looped through a tab on the mold itself and allow the patient to remove it like a tampon. The curvature in the mold allowed the distal third of the mold to sit vertically in the vagina while the proximal portion of the mold would sit horizontally on the levator plate. The mold was curved to a 130° angle to conform with previously published anatomic studies.^8,9^ We maintained the channel through the center of the mold for vaginal drainage throughout our designs. The progression of mold design is shown in Figure 2.

*Bodily. 3D-Printed Vaginal Molds. O&G Open 2025*.

**Fig. 3.**
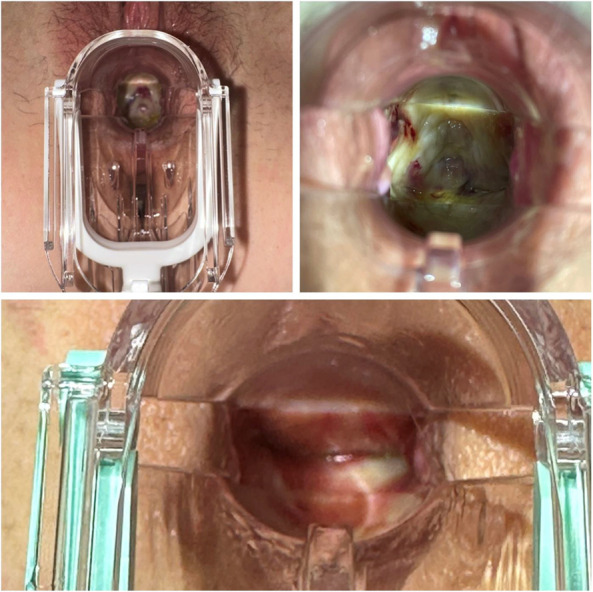
Wound healing at 2 weeks (*top left*), 1 month (*top right*), and 3 months (*bottom*).

At her 2-week follow-up visit (Fig. 3), the patient started hyperbaric oxygen therapy and vaginal estrogen. At 1-month she was enrolled in pelvic floor physical therapy, with emphasis on myofascial release and relaxation (Fig. 3). At her 3-month follow-up visit, she continued to wear the mold overnight and reported only slight discoloration of the mold, which did not affect its integrity (Fig. 3). Six months postoperatively, she was able to have penetrative intercourse without dyspareunia and reported greatly improved quality of life.

*Bodily. 3D-Printed Vaginal Molds. O&G Open 2025*.

## DISCUSSION

Vaginal dilation is the primary treatment for preventing vaginal stenosis after pelvic radiation. Traditional vaginal dilation therapy is an “active” process requiring patients to insert a dilator and maintain pressure for 15–30 minutes multiple times per day or week. Vaginal molds represent a “passive” process in which the patient can insert the mold like a tampon or pessary and continue with their usual activities. The patient in our report used the mold as an insert to maintain vaginal caliber rather than as a dilator.

Kisby et al^10^ and Lim et al^11^ have previously shown success with 3D-printed molds or stents in preventing vaginal stenosis after surgical neovagina creation in cases of vaginal agenesis. Kisby et al, in particular, highlight the benefits of customized, easy-to-use molds; their patient maintained a patent neovagina after 1 year, whereas two previous neovaginas had failed with traditional dilator use.

When these authors created their molds, they used dental resin and thermoplastic polyurethane. We elected to use thermoplastic polyurethane to print our vaginal mold. Its properties, such as its inertness, resilience, and biocompatibility, make it highly desirable for medical applications. Thermoplastic polyurethane has been used successfully in various health care 3D-printing applications, including catheters, stents, and pessaries.^12–14^ We used Ultrafuse TPU 85A Filament due to its compatibility with our 3D printer.

The printing of the mold was completed in 2 hours and 44 minutes and required 27.46 g of Ultrafuse TPU 85A Filament—1.75 mm. The cost of 750 g of thermoplastic polyurethane is approximately $68 or $0.09/g, making the cost of the individual mold $2.47. The cost of the hardware is approximately $1,099 for the Original MK4 Input Shaper with a 0.4 mm nozzle. The Fusion 360 software used requires a license for commercial use, but other free software exists. The PrusaSlicer and PrusaSlicer G-code Viewer software are free to use.

One of the limitations in our design is that we used a fused deposition modeling printer. These printers have a lower resolution and accuracy when compared with other additive manufacturing methods such as selective laser sintering and stereolithography and are not the best option for parts with intricate features or smooth surfaces. Fused deposition modeling printers create small gaps and crevices between the layers of filament, which may result in bacteria and biomaterials being trapped. The advantages of fused deposition modeling printing are that it is inexpensive, easily available, and excellent for prototyping. A future direction for our work would be to use selective laser sintering or stereolithography printers, which rely on the materials being fused with a laser or cured with ultraviolet light to create completely smooth finishes that would be better suited for intravaginal applications. The ideal material for vaginal mold production is not yet clear. Larger prospective studies would be needed to answer this and other questions to facilitate U.S. Food and Drug Administration (FDA) approval.

Additionally, the application and generalizability of this project are limited due to its lack of FDA approval and because not all institutions have access to 3D printers. The patient was counseled that the mold was not FDA approved and would be used in a research capacity. There are FDA-approved, commercially produced molds available that are limited by standardized sizing options.^10,15^ With 3D printing, we were able to modify the mold to increase patient comfort and to have a recess along the anterior portion to accommodate the urethra, allowing the patient to void without having to remove the mold. In the future, vaginal dimensions could be obtained before radiation therapy so that molds could be used prophylactically to prevent vaginal stenosis.

The applications for 3D printing continue to expand, and the technology is readily available and relatively inexpensive. Customized vaginal molds have the advantage of comfort and customization, which overcome the barriers of commercially available dilators and represent an exciting step forward in the treatment and prevention of vaginal stenosis after pelvic radiation.
